# Lactate in contemporary biology: a phoenix risen

**DOI:** 10.1113/JP280955

**Published:** 2021-02-25

**Authors:** George A. Brooks, Jose A. Arevalo, Adam D. Osmond, Robert G. Leija, Casey C. Curl, Ashley P. Tovar

**Affiliations:** ^1^ Exercise Physiology Laboratory Department of Integrative Biology, University of California Berkeley CA USA

**Keywords:** exercise, fibre type, gene adaptation, gluconeogenesis, glycogenolysis, indirect pathway, lactate shuttle, lactate signalling, microbiome, muscle, postabsorptive metabolism, postprandial metabolism, satiety

## Abstract

After a century, it's time to turn the page on understanding of lactate metabolism and appreciate that lactate shuttling is an important component of intermediary metabolism *in vivo*. Cell‐cell and intracellular lactate shuttles fulfil purposes of energy substrate production and distribution, as well as cell signalling under fully aerobic conditions. Recognition of lactate shuttling came first in studies of physical exercise where the roles of driver (producer) and recipient (consumer) cells and tissues were obvious. Moreover, the presence of lactate shuttling as part of postprandial glucose disposal and satiety signalling has been recognized. Mitochondrial respiration creates the physiological sink for lactate disposal *in vivo*. Repeated lactate exposure from regular exercise results in adaptive processes such as mitochondrial biogenesis and other healthful circulatory and neurological characteristics such as improved physical work capacity, metabolic flexibility, learning, and memory. The importance of lactate and lactate shuttling in healthful living is further emphasized when lactate signalling and shuttling are dysregulated as occurs in particular illnesses and injuries. Like a phoenix, lactate has risen to major importance in 21st century biology.

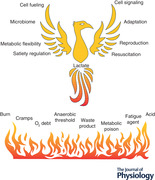

## Graphical abstract

**Abstract figure legend**: Roles of lactate in physiology and metabolism elaborated as: cell fuelling (the preferential use of lactate over other fuel energy substrates such as glucose, fatty and keto acids in cardiac and red skeletal muscle and brain for cognition); cell signalling (the mechanisms by which lactate affects metabolism via effects on cell redox, ROS production, lactylation and covalent binding); microbiome (the role of fermentation in health and disease and the possibility of a gut‐soma lactate shuttle); satiety (the role of lactate on the arcuate nucleus of the hypothalamus and gut‐hypothalamic signalling); adaptation (the role of lactate in expression of glycolytic and metabolic enzymes and insulin sensitivity, the latter by TGF‐β); reproduction (roles of lactate in sperm mitochondrial energetics, developing a favourable microenvironment for blastocysts and secretion of VEGF for uterine angiogenesis), and resuscitation (treatments of dehydration, acidosis, dengue, pancreatitis and hepatitis, myocardial infarction and wound healing).

## Introduction

The story of lactate and its role in physiology and medicine may be a century old, but has changed dramatically in the last three decades (Brooks, 1986, 2002, 2018; Gladden, 2004). No longer conceived of as a dead‐end metabolite, a fatigue agent, or metabolic poison, in contemporary physiology, lactate is seen as a major metabolic intermediate that has wide ranging impacts in energy substrate utilization, cell signalling, and adaptation; simply, lactate is at the fulcrum of metabolic integration (Brooks, 1984, 1986, 2020 *a*). Now, rather than regarded as an oddity of exercise metabolism (Gladden, 2004; Chen *et al*. 2016; Hui *et al*. 2017; Brooks, 2018; Ferguson *et al*. 2018), the presence of lactate shuttling is recognized in fields as diverse as wound healing (Hunt *et al*. 2007), cancer biology (San‐Millan & Brooks, 2017), insulin secretion (Rutter *et al*. 2015), management of sepsis (Garcia‐Alvarez *et al*. 2014 *a*), learning and memory (Suzuki *et al*. 2011; El Hayek *et al*. 2019), and treatment of traumatic brain injury (TBI) (Brooks & Martin, 2014). Hence, in contemporary biology, the role of lactate in metabolism needs to be understood and viewed as a ‘phoenix risen’ (Brooks, 2002, 2018) in contrast to a remnant of (1988) early 20th century biology (Rabinowitz & Enerback, 2020).

As scholars we have our limitations and, as a consequence, in formal education we rely on tradition to a certain extent despite obvious evidence to the contrary. Commencing with work of early 20th century Nobel prize winners (Hill, 1914; Meyerhof, 1920), the role of lactate in metabolism has been misunderstood and perpetuated. Even as their cell and tissue cultures incubated in air often turned acidic overnight, textbook authors (e.g. Lehninger, 1970) perpetuated the myth of oxygen‐limited metabolism giving rise to lactate formation despite the fact that the partial pressure of oxygen in air over their mitochondrial preparations and culture dishes was 3–5 times higher than *in vivo* (Brooks *et al*. 2019). Hence, a reckoning of minds in biology is necessary to understand human and mammalian metabolism in a contemporary context.

Take for instance conflations of terms ‘glycolysis’ and ‘fermentation.’ In mammalian tissues, glycolysis (i.e. conversion of glucose and glycogen to lactate) is very different from aerobic fermentation of sugar to alcohol by yeast, or the production of swamp gases by anaerobic bacteria as occurs in the colon, at wound sites or in rotting (Brooks, 2018, 2020 *c*; Ferguson *et al*. 2018). Conflation of the terms perpetuates the myth of lactate poisoning and thus handicaps understanding of basic biology and its translation. Fortunately, textbook authors are noting a distinction between glycolysis as occurs in animals *in vivo*, and fermentation processes that occur in microbes giving rise to ethanol and foul gases (Urry *et al*. 2020).

## Lactate shuttling: roles of driver and recipient cells

**Figure 1:**
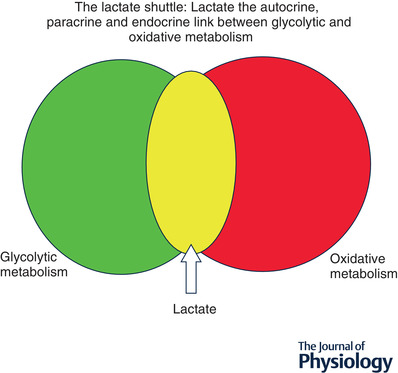
The concept of lactate shuttling between producer (driver) cells and tissues and consumer (recipient) By this mechanism lactate has autocrine‐, paracrine‐ and endocrine‐like influences on metabolism that fulfil at least three purposes. Lactate is: (1) a major energy source; (2) the major gluconeogenic precursor; and (3) a signalling molecule. Revised from Brooks (2018).

Like other metabolites, lactate flux between cells and tissue beds depends on concentration and hydrogen ion (pH) differences. Lactate exchanges between and among cells are facilitated by the presence of cell membrane lactate transport proteins termed monocarboxylate transporters (MCTs) (Garcia *et al*. 1994, 1995); MCTs are bidirectional symporters (Roth & Brooks, 1990*a*,*b*; Brown & Brooks, 1994) sensitive to trans‐stimulation by lactate and hydrogen ion gradients. Hence, we have the concept of glycolytic lactate producing (driver) and lactate consuming (recipient) cells (Fig. 1). From first observations on dog gracilis muscle preparations that showed net lactate release at rest, an increased release when contractions started followed by net lactate uptake as contractions continued (Stainsby & Welch, 1966), the concepts of lactate driver and recipient cells could have been predicted. Subsequently, similar results of net lactate release from resting muscle, followed by increased release and switch to uptake during exercise were confirmed in human muscles (Stanley *et al*. 1985; Bergman *et al*. 1999 *b*). Again, from such results, the presence of lactate driver and recipient cells as part of lactate shuttling could be inferred. Not surprisingly, we now know not only that driver and recipient cells can switch roles depending on conditions, but that some cells can exchange lactate through the interstitium and vascular beds. For instance, during exercise, fast white fibres can provide oxidizable substrate to red, oxidative fibres in the same tissue bed (Baldwin *et al*. 1972; Hooker & Baldwin, 1979). Conversely, postprandial glucose uptake in red fibres can provide substrate to the body corpus as in the glucose paradox (Foster, 1984). Also, working muscle can fuel the beating heart (Gertz *et al*. 1981), brain (Suzuki *et al*. 2011; Glenn *et al*. 2015 *a*; Steinman *et al*. 2016) and provide gluconeogenic substrates to the splanchnic organs (Bergman *et al*. 2000; Gerich *et al*. 2001).

Lactate exchanges among muscle, heart, liver and kidneys are obvious examples of lactate shuttling. However, are other organs involved? Some might argue that the integument is the largest organ in the body, and it is known to produce lactate under sympathetic stimulation (Johnson & Fusaro, 1972). Hence, is there a skin‐muscle lactate shuttle during exercise (Brooks, 2018)? Similarly, the gut, the only organ of fermentation in the body, produces lactate because of the presence of bacteria and dietary fibre in the colon may release lactate into the systemic circulation. Hence, we can speculate on the presence of a gut to soma lactate shuttle (Brooks, 2018). As there are no obvious venous drainage vessels to catheterize, other methods to detect lactate exchange in the integument and gut will need to be found to quantify their roles in lactate shuttling under various conditions.

## Contributions from muscle and exercise physiology (the cell‐cell and intracellular lactate shuttles)

### The cell‐cell lactate shuttle

As is often the case, it is difficult to identify a seminal moment of discovery or publication, but from this perspective, it was results on dog muscles made to contract *in situ* (Stainsby & Welch, 1966). At rest, those highly red, richly perfused muscles always released lactate. Then, at the onset of contractions, lactate release increased, but then switched to net uptake as contractions continued and oxygen consumption rose (*vide supra*). So, in a kernel, we have it, muscle lactate production under fully aerobic conditions followed by uptake as oxygen consumption rises to meet metabolic demand. Subsequently, using NADH fluoroscopy, investigators (Jöbsis & Stainsby, 1968) verified that working muscles were not oxygen limited. Later efforts to study working muscle lactate metabolism (Honig *et al*. 1992) using myoglobin cryomicroscopy again showed ‘aerobic glycolysis’ in working muscle. Importantly, Gladden and associates determined effects of exercise intensity, blood lactate concentration, O_2_ consumption and pH on lactate production and disposal using arterial‐venous differences ((a‐v)), blood flow and ^14^C‐lactate measurements on resting and contracting canine muscles *in situ* (Gladden, 1991; Gladden *et al*. 1994; Kelley *et al*. 2002). In their studies investigators showed lactate turnover (production and disposal) in resting and contracting, fully oxygenated and circulated canine muscle (for reviews see Gladden, 2004; Rogatzki *et al*. 2015).

The advent of radiotracers and their use in physiology and metabolism soon showed continuous lactate production under fully aerobic conditions in dogs (Depocas *et al*. 1969) and rats (Freminet *et al*. 1974; Donovan & Brooks, 1983), with disposal during exercise by oxidation and conversion to glucose (Brooks & Donovan, 1983). Moreover, in studies on rats recovering from exercise, the so called ‘oxygen debt’ (now referred to as EPOC: Gasser & Brooks, 1984) period showed oxidative disposal (4/5) predominating over gluconeogenesis and glyconeogenesis (1/5) (Brooks *et al*. 1973; Brooks & Gaesser, 1980; Gaesser & Brooks, 1980). Compared to the classic results of Meyerhof on isolated frog muscle preparations *ex vivo* (Meyerhof, 1920), results on rats *in vivo* upended the long‐held idea that after exercise 4/5 of the lactate produced during contractions was restored to muscle glycogen *in situ*.

Initial efforts to study lactate‐glucose interactions in humans involved (a‐v) and blood flow measurements across working muscle and splanchnic tissue beds (Ahlborg & Felig, 1982; Wahren & Ekberg, 2007). Such experiments are highly invasive and difficult to conduct and interpret. Nonetheless, the significance of the technologies employed and lessons learned from those difficult experiments showing inter‐organ metabolite exchanges in resting and exercising humans are noteworthy.

The advent of stable, non‐radioactive tracers and their use in physiology and metabolism soon replicated what was shown in mammalian models (Stanley *et al*. 1985, 1986; Mazzeo *et al*. 1986). Subsequently, combined use of (a‐v) differences and blood flow, as well as isotopic tracers, showed simultaneous lactate production and oxidative disposal within resting and working human skeletal muscles (Stanley *et al*. 1986; Bergman *et al*. 1999 *b*). More recently, the same technologies have been employed to show lactate turnover (production, net uptake and oxidative disposal) in the heart (Gertz *et al*. 1981, 1988; Bergman *et al*. 2009 *b*) and brain (Glenn *et al*. 2015 *a*).In their studies of muscle lactate metabolism Richardson and colleagues made ingenious use of repeated studies of muscle net balance ((a‐v)Δ and blood flow) and nuclear magnetic resonance (NMR) to determine intracellular $P_{O_{2}}$ from the myoglobin spectrum (Richardson *et al*. 1998).

Their results showed lactate production in, and release from, resting and exercising muscle when intracellular $P_{O_{2}}$ was significantly above the critical mitochondrial oxygen tension as previously determined (Wilson *et al*. 1988). Similarly, with their MRS experiments on rat heart preparations, Kreutzer and Jue demonstrated myocardial lactate formation above an intracellular $P_{O_{2}}$ that was well above the threshold for decreased ATP production (0.80 mmHg) (Kreutzer & Jue, 1995). Thus, while there is no singular value that would imply a threshold for increased lactate production in human muscle or rat heart, it is certain that lactate is formed under fully aerobic, normoxic conditions (Connett *et al*. 1990).

Complimentary to determinations of lactate disposal via oxidation were those determining lactate disposal via gluconeogenesis in resting postabsorptive humans. Hence, it is fair to state that while most lactate disposal is accomplished via oxidation, with a minority of lactate disposal accomplished via gluconeogenesis under postabsorptive resting conditions, lactate is the most important gluconeogenic precursor during rest and exercise (Stanley *et al*. 1988; Bergman *et al*. 2000; Gerich *et al*. 2001)

**Figure 2:**
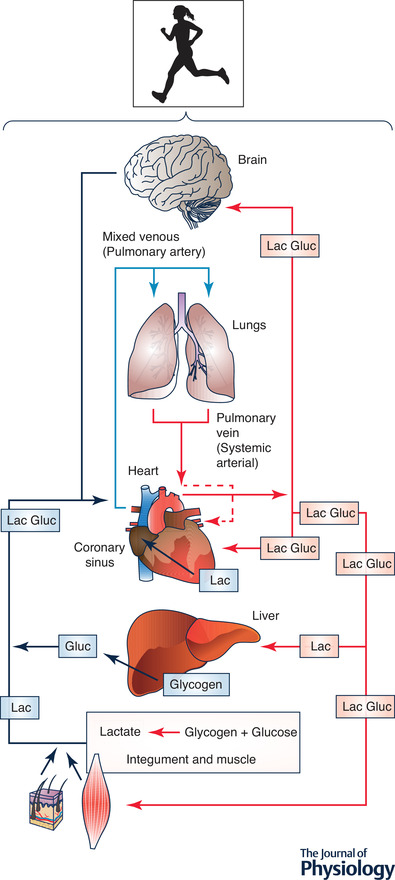
Illustration of lactate shuttling during exercise (the cell‐cell lactate shuttle) Lactate released from muscles, skin and other driver cells provides energy for working muscles (Stanley *et al*. 1986; Bergman *et al*. 1999 *b*), heart (Gertz *et al*. 1981, 1988; Bergman *et al*. 2009 *b*) and brain (Glenn *et al*. 2015 *a*). Moreover, lactate released from working muscles (Stanley *et al*. 1986; Bergman *et al*. 1999 *b*) and other driver cells such as adipose tissue is the major gluconeogenic precursor (Stanley *et al*. 1988; Bergman *et al*. 2000) and brain fuel, even after injury when lactate supplementation may be efficacious (Brooks & Martin, 2014). Revised from Brooks (2018).

In sum, the above‐cited work shows continuous aerobic (*not* oxygen‐limited) lactate turnover (production and disposal) in humans and mammalian model systems. Further, the work shows two of three features of the lactate shuttle – lactate production in driver cells and disposal in recipient cells and tissues; signalling being a third feature of the lactate shuttle (Brooks, 2000, 2002, 2018). Hence, we now realize that lactate produced in working muscle is a major fuel energy source (Stanley *et al*. 1986; Bergman *et al*. 1999 *b*), but also that lactate release from working muscle fuels other organs such as the heart (Gertz *et al*. 1988; Bergman *et al*. 2009 *b*) and brain (Glenn *et al*. 2015 *a*) normally, but also in illness and following injury (Marik & Bellomo, 2013; Brooks & Martin, 2014; Garcia‐Alvarez *et al*. 2014 *b*). Moreover, lactate released from working muscles (Stanley *et al*. 1986; Bergman *et al*. 1999 *b*) and other driver cells such as the integument is the major gluconeogenic precursor (Stanley *et al*. 1988; Bergman *et al*. 2000) (Fig. 2).

### The intracellular lactate shuttle (ICLS)

The above‐cited studies using infused ^13^C‐lactate and observing ^13^CO_2_ excretion from resting and working muscles show simultaneous lactate uptake, production, and oxidation *in vivo* (Stanley *et al*. 1986; Bergman *et al*. 1999 *b*). However, those studies do not provide information on the intracellular path of lactate oxidation. Hence, studies on isolated mitochondria (Brooks *et al*. 1999 *c*, 1999 *d*; Dubouchaud *et al*. 2000; Hashimoto *et al*. 2006, 2008; Atlante *et al*. 2007; Passarella *et al*. 2008, 2014) and muscles (Park *et al*. 2015) were called for.

To understand mitochondrial lactate oxidation *in vivo*, one needs to appreciate that the cellular respiratory apparatus is composed not of discrete vesicles (i.e. mitochondria), but rather an extensive network, a mitochondrial reticulum (Bakeeva *et al*. 1978; Kirkwood *et al*. 1986, 1987) that extends from the sub‐sarcolemmal domain to deep within fibres, that represents an ‘energy highway’ (Glancy *et al*. 2015). To oxidize energy products of glycolysis, the reticulum contains both lactate (mMCT) (Brooks *et al*. 1999 *c*) and pyruvate (mPC) transporters (Bricker *et al*. 2012; Herzig *et al*. 2012). Importantly, because the product of glycolysis is lactate, not pyruvate (Rogatzki *et al*. 2015), mitochondrial uptake and oxidation far exceeds that of pyruvate. Hence, studies of muscle lactate oxidation led to discovery of the mitochondrial lactate oxidation complex (mLOC) *in vivo*.

In terms of functionality, the mLOC contains several essential components of lactate oxidation: an MCT, its membrane chaperone Basigin (BSG or CD147), lactate dehydrogenase (LDH), and cytochrome oxidase (COx) as seen in muscle (Hashimoto *et al*. 2006), liver (De Bari *et al*. 2004; Passarella *et al*. 2014), and brain (Hashimoto *et al*. 2008), and various model systems such as brain slices (Schurr, 2008), primary neuronal cultures (Atlante *et al*. 2007; Hashimoto *et al*. 2008), normal breast and transformed breast cancer cells (Hussien & Brooks, 2011), and tumours (Sonveaux *et al*. 2008). Colocalization of MCTs, LDH and COx has been seen in muscles (Hashimoto *et al*. 2005, 2007, 2013), brain (Hashimoto *et al*. 2008), lung (Johnson *et al*. 2012), and cancer cells (Hussien & Brooks, 2011).

Confirmation of the preference of mitochondrial lactate over pyruvate oxidation comes from studies of hyperpolarized lactate in muscles *in situ* (Park *et al*. 2015). So far as mitochondrial pyruvate transport and oxidation are concerned, it is known that the putative mPC colocalizes with mitochondrial MCT1 in L6 myocytes. In preliminary studies, colocalization analysis of mMCT1 and mPC1 using Imaris software revealed that both MCT1 and mPC are co‐localized to the mitochondria (*r* ^2^ = 0.8) (Brooks, 2020*c*). However, because muscle lactate concentration exceeds that of pyruvate by one (10×) to two (200–400×) orders of magnitude in resting and exercising human muscles, respectively (Henderson *et al*. 2004), lactate oxidation is dominant *in vivo*.

As will be discussed in more detail later, an important feature of lactate shuttling is that it affects cell redox in both driver and recipient cells, and between cell compartments. At present there is discussion over whether the ICLS functions independently of or cooperatively with longer established malate‐aspartate and glycerol phosphate shuttles (Kane, 2014). Given redundancies in physiological control systems, it may be that cytosolic‐to‐mitochondrial shuttles work in parallel to manage the lactate load and balance cell redox during exercise and other conditions. Using CRISPR knockout technology in mouse or other mammalian models may provide missing data on the relative uses of specific intracellular redox shuttles under particular physiological circumstances (Kane, 2014).

### Lactate shuttling as a basis for the lactate threshold in physical exercise

It has been half a century since introduction of the concept of oxygen‐limited metabolism producing an ‘anaerobic threshold’ (AT), that is, O_2_ limited metabolism during exercise of a particular intensity. The subject has been recently reviewed (Poole *et al*. 2021). With 1541 citations (Web of Science, 27th September, 2020) the paper of (Wasserman & McIlroy, 1964) is the eighth most cited paper of all time in the *Journal of Applied Physiology*. As recited in the recent review (Poole *et al*. 2021), in a letter from Karlman Wasserman to this author, the concept of an ‘anaerobic threshold’ was based on the assumption that ‘failure of the heart to transport O_2_ adequately would result in lactic acidosis’, an event that would trigger ventilatory compensation. Subsequently, concepts of anaerobic, lactate, ventilatory and other related thresholds (e.g. OBLA) are pervasive in the literature and have represented key concepts in basic and applied physiology, sports and pulmonary medicine. These thresholds are determined by turnpoints in the rise in blood lactate concentration, pulmonary minute ventilation, and heart rate during graded exercise. In a PubMed (National Library of Science) search on 20th October, 2020 the term ‘anaerobic threshold’ elicited 5902 citations. In their review, Poole *et al*. explain that while the basic tenant of the AT (i.e. oxygen‐limited metabolism) in exercise is no longer acceptable, use of the ‘gas exchange threshold’ (GET) in graded exercise possesses significant importance for cardio‐pulmonary and other types of evaluation in patients (Poole *et al*. 2021).

In sum, intracellular lactate disposal is accomplished by a transport mechanism for direct mitochondrial uptake and oxidation that involves a mLOC which probably works in parallel with malate‐aspartate, glycerol‐phosphate and other shuttles to balance cell redox and manage the lactate load from glycolysis under normal and stressful conditions (Brooks, 2020*c*).

## Contributions from studies of postprandial metabolism: the glucose paradox (indirect pathway of liver glycogen synthesis) and the postprandial lactate shuttle

### The glucose paradox

**Figure 3:**
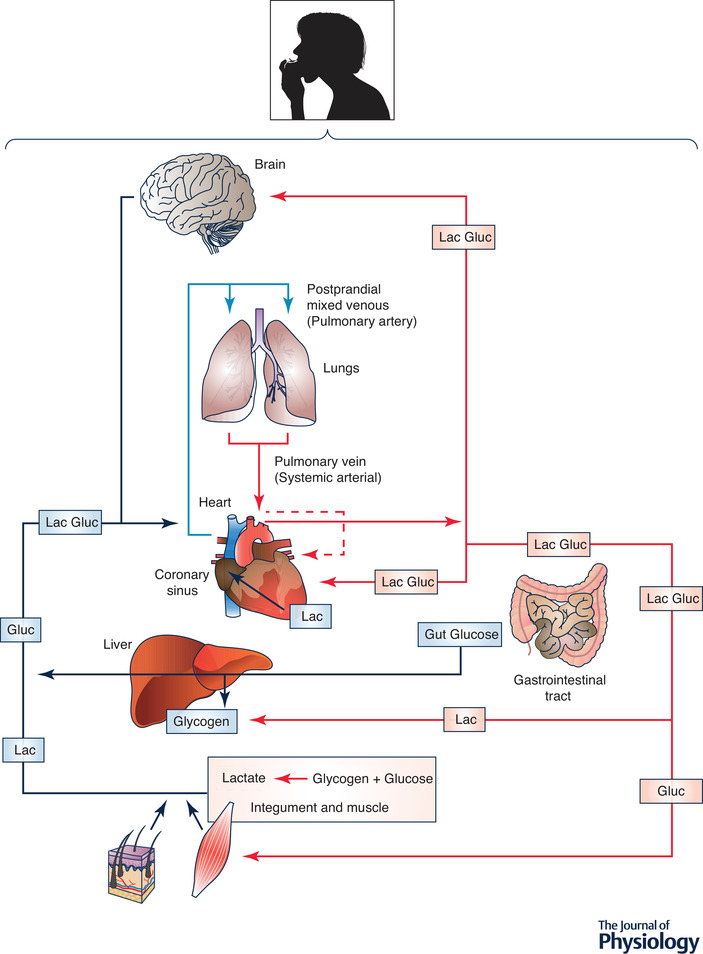
Illustration of lactate shuttling after consuming dietary carbohydrate: the postprandial period (the postprandial lactate shuttle) Depending on liver glycogen content some dietary glucose bypasses liver and enters the systemic circulation. From there glucose is taken up by non‐contracting muscles, particularly red and intermediate fibres (James *et al*. 1985, 1986). Glycolysis in these and other driver tissues results in lactate release into the central venous circulation and uptake by the recipient liver from the arterial circulation. Paradoxically, this circuitous, ‘indirect pathway,’ is preferred over glucose for hepatic glycogen synthesis (Foster, 1984). In contrast, glucose from dietary consumption or digestion or carbohydrate in the GI tract can be taken up by liver on the first circulatory pass for ‘direct’ glycogen synthesis. Again as indicated in Fig. 2, lactate released from driver cells and tissues supports cerebral metabolic needs (Glenn *et al*. 2015 *a*,*b*) and functions such as glutamatergic signalling (Pellerin & Magistretti, 1994). Similarly, systemic lactate and fatty acids from digestion serve as an energy source for the heart (Bergman et al. 2009a,b). Figures 2 and 3 illustrate the extent of lactate shuttling under diverse conditions.

Studies of postprandial glucose metabolism on rodent models and humans show what has been termed the ‘glucose paradox,’ or ‘indirect pathway of hepatic glycogen synthesis’ (Foster, 1984). This concept states that dietary glucose released into the hepatic portal vein initially bypasses the liver and goes to the periphery, where glycolysis converts glucose to lactate that is subsequently released into the central venous circulation and taken up from the arterial circulation by liver for glycogen synthesis. This, paradoxical, ‘indirect’ pathway of hepatic glycogen synthesis is to be contrasted with the ‘direct’ pathway in which dietary glucose from the gut is taken up and converted to liver glycogen on first circulatory pass (Fig. 3).

The initial concept developed from studies on lab animals has been replicated in human subjects showing both indirect and direct liver glycogen synthesis in healthy, postprandial humans. However, the balance of indirect and direct glucose conversion appears to be species related. Results of Gerich and colleagues in human subjects confirm that glycolysis is the main initial postprandial fate of glucose, accounting for ≈66% of overall disposal, while oxidation and storage each accounted for ≈45%. However, the majority of hepatic glycogen synthesis in postprandial humans (≈73%) was formed via the direct pathway (Woerle *et al*. 2003). In the near future it should be possible to better understand the issue of influences of diet and other factors on the balance of direct *vs*. indirect liver and muscle glycogen synthesis in humans using ^13^C‐tracers and magnetic resonance spectrometry (Stender *et al*. 2020).

While considered to be a homogenous ‘organ system,’ muscle is in fact a heterogeneous tissue containing different types of muscle fibres, and circulatory and connective tissue networks, all with different metabolic characteristics (Barnard *et al*. 1970, 1971). Postural muscles (e.g. soleus, erector spinae) are alternatively termed intermediate (i.e. pink, not red or white), or Type I fibres. In many species deep vastus and lateral gastric are bright red and termed red or Type IIA fibres. In contrast white, fast twitch fibres are termed Type IIX (in humans) or IIB (in rodents). Hence, results of the above‐cited studies on the glucose paradox are complimented by results of studies showing greater postprandial perfusion and glucose uptake in muscles containing predominantly oxidative Type I and IIA fibres than in muscles containing predominantly Type IIB/X fibres (James *et al*. 1985, 1986). Redundancy of terminology in science is confusing, but the glucose paradox can also be thought of as a postprandial lactate shuttle.

### Phasic lactate shuttling: lessons from the heart

Tracer studies allow us to know that the heart produces lactate even as it acts as a lactate sink displaying net uptake from the arterial circulation (Gertz *et al*. 1988; Bergman *et al*. 2009 *b*). Assessing cardiac metabolism in humans is difficult at best and is complicated by the cardiac cycle. If it is true that even in a healthy heart systole interrupts coronary blood flow, especially in the endocardium, then the presence of a phasic intra‐cardiac lactate shuttle is indicated. Assuming a resting heart rate of 60 bpm, then systole followed by diastole occurs once per second. This could mean that there occurs 200 ms of stopped flow during the isometric phase of systole which is powered by glycolysis, followed by 800 ms of diastole for oxidative recovery and lactate clearance. Then heart rate increases during exercise, and time for the T‐P interval decreases, so also does the time for restoration of myocardial blood flow and intra‐cardiac oxidative metabolism decrease. Again, as with the hypothesis of gut lactate shuttling, the presence of an intra‐cardiac lactate shuttle would be difficult to prove because even coronary sinus sampling would be insufficient to determine phasic events. Hopefully in the future, hyperpolarized MRS (Park *et al*. 2015) or other technologies will allow for detection of lactate production, net uptake and oxidative disposal within a cardiac cycle.

For the present, perhaps the best evidence of a phasic, intra‐cardiac lactate shuttle may come from the lactate threshold/turn point field in which a steep rise in blood lactate occurs at 90% of maximal heart rate (Hofmann *et al*. 1994). Even in the healthy hearts of athletes with high heart rates, there simply may be inadequate time for intra‐myocardial lactate disposal plus a burden of systemic blood lactate clearance.

## Can the splanchnic bed be a source of lactate?

### Upper gut: the liver

Classically, the liver removes lactate from the circulation and converts it to glucose for release into the systemic circulation (Cori & Cori, 1946), or converts lactate to liver glycogen (Nilsson & Hultman, 1974). But, can the liver, or splanchnic bed as a whole contribute lactate to the systemic circulation? Evidence for splanchnic lactate production is sparse.

Hepatic release from dog liver under glucagon stimulation was not seen (Wasserman *et al*. 1984). However, after glucose ingestion in rats with indwelling portal vein catheters, a porto‐peripheral lactate gradient was present, reflecting the production of lactate in or by the intestine (Smadja *et al*. 1988). Unfortunately, because studies on humans would involve hepatic portal vein catheterization, an invasive procedure not known to be associated with any disease state, and hence probably ethically unjustified, we know of no report of hepatic or splanchnic net release during postprandial exercise. However, because the phenomenon of splanchnic lactate release has not been observed, it doesn't necessarily follow that the phenomenon fails to exist.

In addressing the issue of upper gastrointestinal (GI) lactate production in humans, consideration of the carbohydrate energy form may be helpful. Using combinations of glucose, fructose and lactate tracers to evaluate the use of oral carbohydrate energy sources in sports drinks investigators in the Tappy lab (Lecoultre *et al*. 2010; Theytaz *et al*. 2014) observed carbon atoms from an orally ingested fructose tracer appearing in the systemic circulation as labelled lactate. Hence, counter to classic thought, there is evidence for postprandial splanchnic lactate release in humans following the ingestion of one carbohydrate energy source, fructose.

In sum, like the event of physical exercise in which lactate plays a prominent role in energy substrate production and disposal, lactate also plays an important role in carbohydrate distribution and disposal after eating. Hence, whether after a meal when glycogen storage is prominent, or during post‐absorptive exercise when glycaemia is supported by hepatic glycogenolysis and gluconeogenesis, lactate plays important roles in carbohydrate (CHO) energy substrate distribution (Brooks, 2020*b*). The issue of GI lactate production is addressed further in the section on the microbiome below.

### Lower gut: the microbiome

The possibility of participation of the gut microbiome in lactate shuttling and a gut‐soma lactate shuttle has previously been suggested (Brooks, 2018, 2020 *a*), but we are in the early stages of discovery. Support for the idea is to be found in diverse sources. From epidemiology, we know that regular physical exercise is beneficial for reducing risks of many common cancers including those of the colon (Powell *et al*. 2018). Also, from nutrition science we know that pre‐ and pro‐biotic dietary components favourably affect gut fermentation and health (Hill *et al*. 2014) and data indicate relationships between microbiota and the prevalence of insulin resistance and metabolic syndrome (Vrieze *et al*. 2012). The concentration of lactate in human faeces is relatively low (< 5 mm) because of the presence of bacteria that convert lactate to butyrate (Duncan *et al*. 2004). More recently, we have learned that physical exercise and physical fitness encourage diversity of species including those that act to convert lactate to butyrate (Estaki *et al*. 2016, 2020). Recently also, the presence of ‘performance‐enhancing’ gut microbes, members of the genus *Veillonella*, in the stools of marathon runners (Scheiman *et al*. 2019) have been found. Unfortunately, those investigators were unaware of the importance of lactate shuttling during exercise, and did not consider a scenario in which the gut supplies lactate, a fermentation product that is exported via sodium‐mediated monocarboxylate transporters (sMCT) (Coady *et al*. 2004; Teramae *et al*. 2010), thus supporting athletes’ efforts by fuelling working muscles as opposed to clearing lactate from the circulation.

## Lactate controls lipid metabolism: the role of lactate in metabolic flexibility and the crossover concept

Lactate from working muscle (Bergman *et al*. 1999 *b*) or other tissues such as the integument (Johnson & Fusaro, 1972) has short‐term effects on lipid metabolism by downregulating both lipolysis and mitochondrial free fatty acid entry and oxidation. In contrast, chronic exercise exposure, as with endurance training, improves capacities to maintain glycaemia and lipid oxidation by improving the ability to clear lactate by oxidation (Bergman *et al*. 1999 *b*) and gluconeogenesis (Bergman *et al*. 2000), as well as acting as a pseudo‐myokine (Takahashi *et al*. 2019) (*vide infra*). The role of lactate in affecting energy substrate partitioning in exercise and other conditions is imbedded in concepts of ‘crossover’ (Brooks & Mercier, 1994) and metabolic flexibility (Kelley *et al*. 1999; Goodpaster & Sparks, 2017; San‐Millan & Brooks, 2018). In the case of lactate and lipolysis in adipose tissue, during hard exercise inverse relationships between blood lactate and plasma free fatty acid concentration [FFA] in humans (Brooks & Mercier, 1994) and other mammals (Issekutz & Miller, 1962; Rodahl *et al*. 1964) have long been recognized. Also, lactate infusion into running dogs caused the circulating [FFA] to decline (Issekutz & Miller, 1962; Gold *et al*. 1963; Miller *et al*. 1964). In those investigations an effect of lactate on circulating [FFA] could be clearly observed, but whether the mechanisms involved hydrogen ions or lactate anions was not assessed.

The mechanism by which lactataemia suppresses circulating FFAs is now known to be due to suppression of adipose lipolysis by lactate binding to hydroxycarboxylic acid receptor 1 (HCAR‐1), formerly known as G‐protein coupled receptor 81 (GPR‐81), independently of changes in pH (Cai *et al*. 2008; Ge *et al*. 2008; Liu *et al*. 2009; Ahmed *et al*. 2010). Signalling effects of lactate on HCAR‐1 are further discussed below.

### Lactate and pyruvate as inhibitors of mitochondrial β‐oxidation

When glycolysis is accelerated during muscle contraction, lactate (L) and pyruvate (P) concentrations rise and increase the L/P ratio, (Bergman *et al*. 1999 *b*; Henderson *et al*. 2004) reducing cytosolic redox (Brooks, 2020*a*). At rest, the L/P ratio in muscle and venous effluent from a muscle bed approximates 10, but the ratio rises more than an order of magnitude (≥ 30×) during moderate intensity exercise (Henderson *et al*. 2004). By mass action the monocarboxylate pair floods into the mitochondrial reticulum (Saddik *et al*. 1993; Brooks *et al*. 1999 *b*; Passarella *et al*. 2008), giving rise to acetyl‐CoA and, thereby, malonyl‐CoA formation. The rise in malonyl‐CoA inhibits the entry of activated FFAs into the mitochondrial matrix by inhibiting carnitine‐palmitoyl transferase‐1 (CPT1) (McGarry *et al*. 1977; Saddik *et al*. 1993). As well, the effect of glycolysis driving an accumulation of acetyl‐CoA downregulates β‐ketothiolase, the terminal and rate‐limiting enzyme of the mitochondrial β‐oxidation pathway. Hence, by mass action, allosteric binding and effects on cell redox, lactate acts to prevent entry of activated fatty acids into the matrix of the mitochondrial reticulum, thus limiting β‐oxidation of fatty acid derivatives.

## Lactate shuttling and metabolic signalling

The effects of increases in cell work and lactate production on redox, reactive oxygen species (ROS), allosteric binding, and histone lactylation have been recently reviewed (Brooks, 2020*a*). Briefly, cell work leads to lactate production and changes in the cellular lactate/pyruvate ratio (Henderson *et al*. 2007) that accompanies changes in the cellular NAD^+^/NADH ratio and subsequent metabolic and regulatory effects in driver and recipient cells (*vide supra*). Importantly also, additional lactate signalling mechanisms have been revealed.

### Cell redox

As described above, muscle contraction causes major changes in the L/P and NADH/NAD^+^ ratios. In driver cells that change allows glycolysis to proceed via providing NAD^+^ for GAPDH, but also by reducing pyruvate to lactate in the cytosol, which creates a driving force for mitochondrial substrate oxidation. In contrast, in recipient cells uptake of lactate raises the NADH/NAD^+^ ratio, thus downregulating glycolysis. This preferential use of lactate over endogenous cellular energy sources reduces oxidation of glucose in muscle (Miller *et al*. 2002 *a*,*b*) and heart (Wisneski *et al*. 1987; Gertz *et al*. 1988; Bergman *et al*. 2009 *a*).

Moreover, aside from the major effects of muscle contraction and glycolysis on energy substrate partitioning, glycolysis leading to lactate production and resulting change in cytosolic NADH/NAD^+^ also effects the status of other redox couples such as the ratio of reduced to oxidized glutathione (GSH/GSSG) (Gohil *et al*. 1988; Viguie *et al*. 1993).

### ROS

Lactate can raise cellular production of ROS via mitochondrial respiration (Powers *et al*. 1999; Passarella *et al*. 2008) and non‐enzymatically via lactate‐iron interactions that are capable of generating ROS (Wagner *et al*. 2004). Another way in which lactate can affect ROS production is via generation of hydrogen peroxide (H_2_O_2_) (Passarella *et al*. 2008). The latter mechanism involves generation of H_2_O_2_ via a flavine‐dependent lactate oxidase located in the mitochondrial intermembrane space (de Bari *et al*. 2010).

### Sirtuins

Sirtuins are deacetylases regulated by the equilibrium between nicotinamide (NAM) and NAD^+^. Sirtuin activation is accomplished via changes in cell redox (i.e. the NAD^+^/NADH) through the concentration of NAM and the activity of the enzyme NAM phosphoribosyl transferase (Nampt). Many investigators are concerned with how the subtle changes in cell redox affect cell homeostasis as occur in apoptosis, inflammation, and other processes. However, more obvious are changes in cell redox with increments in cell work as occur in exercise. At present, data are lacking on the change in Nampt activity in working muscle or non‐working recipient tissues in which changes in NAD^+^ and NADH concentrations are likely to affect sirtuin regulation *in* vivo.

### Hydroxycarboxylic acid receptor 1 (HCAR‐1)

The mechanism by which lactataemia suppresses circulating FFAs is now known to be due to suppression of adipose lipolysis (Ahmed *et al*. 2010). Independently of pH, lactate inhibits lipolysis in fat cells through activation of HCAR‐1 (*vide supra*). In studies of mouse, rat, and human adipocytes, HCAR‐1 appears to act as a lactate sensor with the inhibitory effect on lipolysis operating through cyclic AMP (cAMP) and cAMP response element binding (CREB) (Ahmed *et al*. 2010; Bergersen, 2015).

### Transforming growth factor beta 2 (TGF‐β2)

The effects of lactate on inter‐organ signalling and dual or multiple effects of lactate signalling is illustrated by the recent discovery that transforming growth factor beta 2 (TGF‐β2) is secreted from adipose tissue in response to lactate released from working muscle (Takahashi *et al*. 2019). Because TGF‐β2 improved glucose tolerance in mice, the authors concluded that exercise training improves metabolic regulation through an inter‐organ (adipose to liver) communication via a ‘lactate‐TGF‐β2 signalling cycle.’ If validated on studies of human subjects, TGF‐β2 could be classified as an ‘adipokine,’ and lactate could be thought of as a ‘pseudo myokine’ and the value of enhancing lactate clearance during exercise by endurance training further emphasized (Donovan *et al*. 1983; Bergman *et al*. 1999 *b*; Messonnier *et al*. 2013). In this context both short‐ and long term‐effects are illustrated because lactate is known to suppress lipolysis in adipose via HCAR‐1 and CREB (*vide supra*).

### Lactylation of histones

Regulation of gene expression by lactylation of 28 lysine residues on histones has been demonstrated (Zhang *et al*. 2019). The addition of lactate in addition to phosphate, methyl and acetyl tags to histones is yet another epigenetic way by which the genome and intermediary metabolism are regulated. As noted above, lactate release from driver cells into the circulation, as occurs in physical exercise and other stressful conditions, has the potential for epigenetic modifications in diverse cells around the body during and after physical exercise.

### Lactate, endoplasmic reticulum Mg^2±^ release and mitochondrial function

Recently, Daw *et al*. in the Madesh lab (Daw *et al*. 2020) showed that lactate is an activator of Mg^2+^ release from endoplasmic reticulum (ER). ER Mg^2+^ release facilitates mitochondrial Mg^2+^ (mMg^2+^) uptake, a process facilitated by magnesium lactate transporter 2 (Mrs2). Investigators observed that a surge in mitochondrial Mg^2+^ uptake promotes inflammation and organ failure by interfering with mitochondrial energetics. In contrast, suppression of the mMg^2+^ surge alleviates inflammation‐induced multi‐organ failure. Together, these findings reveal that lactate mobilizes intracellular Mg^2+^ and links the mMg^2+^ transport machinery with major metabolic feedback circuits and mitochondrial bioenergetics (Daw *et al*. 2020). Hence, on the basis of the work by Daw *et al*., it is apparent that, in addition to mass action, redox control, and ROS generation, lactate may affect mitochondrial energetics in other ways such as by Mg^2+^ release from endoplasmic reticulum.

The purported effects of lactate signalling HCAR‐1, TGF‐β2, Nampt, mMg^2+^ and lactylation of histones observed in rodent and cell models await validation in humans. However, for the present, it is certain that lactate both inhibits lipolysis and mitochondrial FFA oxidation in the short‐term and in the long‐term stimulates mitochondrial biogenesis and glucose tolerance and lipid oxidation in *in vivo* (Takahashi *et al*. 2019). Therefore, the new results are encouraging as the signalling patterns are consistent with what is known about the roles of lactate in physiology and metabolism.

## A plethora of lactate shuttles and diverse roles in physiology and metabolism

Since recognition of the presence of lactate shuttling within and among various cells, tissues and organs such as muscle, heart and liver (Brooks, 1984, 1985, 1986, 2002, 2018), the concept has been extended to include other cells, tissues and organs such as brain (Pellerin *et al*. 1998; Liu *et al*. 2017), lung (Johnson *et al*. 2011, 2012), sperm (Storey & Kayne, 1977; Boussouar & Benahmed, 2004), adipose tissue (Cai *et al*. 2008; Liu *et al*. 2009; Ahmed *et al*. 2010), and peroxisomes (McClelland *et al*. 2003). The presences of multiple lactate shuttles with diverse functions in physiology has been recently summarized (Brooks, 2009), but for completeness the following are mentioned.

### The astrocyte‐neuron lactate shuttle and brain functioning

Lactate shuttling between astrocytes and neurons linked to glutamatergic signalling was recognized by Pellerin, Magistretti and colleagues who used the term ‘astrocyte‐neuron lactate shuttle’ (ANLS) (Magistretti *et al*. 1999). Since its introduction and further exposition (Pellerin & Magistretti, 2012), the concept has remained controversial (Patel *et al*. 2014). However, subsequent studies show wide expression of brain MCTs (Pellerin *et al*. 2005) and support the presence of an ANLS.

While cerebral lactate flux may be related to glutamatergic signalling (Pellerin & Magistretti, 1994), there are other processes that are related to lactate shuttling. For instance, in health and after injury (Glenn *et al*. 2015 *a*), as well as during physical exercise (van Hall *et al*. 2009; Hashimoto *et al*. 2018), the brain takes up and oxidizes lactate from the systemic circulation. In such cases, the lactate shuttle concept holds true and explains the exchange between muscle lactate driver and brain recipient cells.

Characteristics of cellular lactate uptake such as stereo‐specificity, concentration and pH dependence, saturation, trans‐stimulation and inhibition by competitive and non‐competitive inhibitors were recognized early on (Watt *et al*. 1988; Roth & Brooks, 1990*a*,*b*; Brown & Brooks, 1994). Since MCT isoforms were cloned and sequenced (Garcia *et al*. 1994, 1995; Price *et al*. 1998) and then studied *in vitro*, it has been appreciated that MCTs facilitate movement of monocarboxylates other than lactate (e.g. pyruvate, β‐hydroxybutyrate and acetoacetate) across biological membranes. However, for at least two sets of reasons, these other monocarboxylates are of lesser physiological importance in terms of carbon‐energy transfer. First, other monocarboxylates do not compete as well as lactate for exchange by MCTs because of Michaelis‐Menten kinetic properties (Roth & Brooks, 1990*a*,*b*). Secondly, physiological levels of lactate exchange competitors are far less abundant (Zinker *et al*. 1990; Bergman *et al*. 1999 *b*; Henderson *et al*. 2004). Importantly, from a physiological standpoint, MCTs do not create the conditions for lactate exchange; rather, MCTs facilitate carbon exchange between loci of driver processes such as glycolysis and glycogenolysis and recipient loci where processes such as mitochondrial respiration and gluconeogenesis are responsible for lactate consumption (Brooks, 1984, 2002). Hence, it is appropriate to recognize the importance of MCTs in cerebral lactate shuttling in cognition, learning, memory and other executive functions (Holloway *et al*. 2007; Suzuki *et al*. 2011; Steinman *et al*. 2016; Hashimoto *et al*. 2018; El Hayek *et al*. 2019).

### Lactate and inflammation

Other than acknowledging its complexity, the role of lactate in promoting or suppressing inflammation is incompletely understood at present. As mentioned above, Daw *et al*. have provided evidence that lactate‐induced Mg^2+^ release from ER affects mitochondrial energetics and could be involved in cell death and organ systems failure (Daw *et al*. 2020). In contrast, others have provided evidence that ‐lactate containing solutions have efficacy as resuscitation fluids for use in a variety of conditions including metabolic acidosis (Brooks, 2018), acute pancreatitis (Wu *et al*. 2011; Hoque *et al*. 2014), hepatitis (Hoque *et al*. 2014), sepsis (Garcia‐Alvarez *et al*. 2014 *b*) and dengue fever (Somasetia *et al*. 2014). In terms of mechanism, Hoque and colleagues (Hoque *et al*. 2014) found that lactate binding to HCAR‐1 downregulates Toll like receptor induction of the pyrin domain‐containing protein 3 (NLRP3) inflammasome and production of IL1beta, via Arrestin beta 2 (ARRβ2) and HCAR‐1, was the mechanism by which lactate suppressed inflammation in patients with acute organ injury. Because of the above, the science and translation of lactate shuttling in inflammation needs further attention.

### The peroxisomal lactate shuttle and β‐oxidation

Peroxisomes are cellular organelles found in virtually all eukaryotic cells that are involved in catabolism of substances such as very long chain (i.e. C22 and longer) fatty acids, branched‐chain fatty acids, d‐amino acids, polyamines, and reactive oxygen species such as hydrogen peroxide (H_2_O_2_) (Gladden, 2004). While it was known that β‐oxidation of very long‐chain fatty acids occurred in mammalian peroxisomes (Lazarow & De Duve, 1976), in the absence of enzyme systems such as exist in the mitochondrial matrix, it was not understood how β‐oxidation could occur in peroxisomes. Key findings were those of Baumgart *et al*. (1996), who showed the presence of peroxisomal LDH isoforms, and McClelland *et al*. (2003), who confirmed the presence of peroxisome LDH and further demonstrated that rat liver peroxisomal membranes contained MCT1 and MCT2. From there, it was possible to hypothesize that peroxisomal redox control necessary to support β‐oxidation involved a lactate‐pyruvate shuttle. Discovery of the peroxisomal lactate shuttle involving pyruvate‐lactate conversion linked to changes in the NADH/NAD^+^ redox couple emphasizes the critical importance of redox changes in all forms of lactate shuttles.

### Spermatogenic and Sertoli‐germ cell lactate shuttles

It has been known for decades that mammalian spermatozoa use lactate as an aerobic energy source (Storey & Kayne, 1977). Noteworthy also is that in their seminal papers on the discovery of MCT1 and MCT2 Garcia *et al*. (1994, 1995) observed high expression of MCT1 in sperm heads as they entered the epididymis, followed by lower expression of MCT1 expression as sperm coursed through the epididymis. It was also noted that lactate stimulates respiration in ejaculated bovine sperm (Halangk *et al*. 1985), and further that lactate maintains bovine sperm motility when maintained *ex vivo* (Inskeep & Hammerstedt, 1985). By analogy, just as fast twitch‐glycolytic driver muscle fibres fuel adjacent slow twitch‐oxidative muscle fibres, Sertoli cells in testes secrete lactate to fuel sperm motility *in vivo*. In effect then, the Sertoli‐sperm cell relationship is the primal demonstration of cell‐cell lactate shuttling. In sperm, the mitochondrial reticulum is elaborate, large and spiral shaped, located at the midpiece (Storey & Kayne, 1977) at the base of the sperm head. The ability of sperm mitochondria to oxidize exogenously supplied lactate (Jones, 1997) is analogous to capacities for mitochondrial lactate oxidation in other tissues (Baba & Sharma, 1971; Kline *et al*. 1986; Brandt *et al*. 1987; Brooks *et al*. 1999 *a*; De Bari *et al*. 2004).

## Not a few, but many observers

Readers may note that the contemporary literature on the role of lactate in exercise physiology and metabolism comes from only a few laboratories (Brooks, 2018; Ferguson *et al*. 2018), but such is not the case if a wider perspective is taken, when it is apparent that support for the biological roles of lactate come from diverse scientific and clinical fields in which solutions to many problems are addressed utilizing a variety of technologies. In addition to the above identified lactate shuttles, the importance of lactate in contemporary biology is illustrated in the following examples.

### The glycogen shunt

Shulman and colleagues used proton (^1^H) and ^13^C magnetic resonance spectroscopy (MRS) to explain the rapid rate of metabolic transitions in brain after stimulation; consequently, a glycogen shunt was proposed (Shulman *et al*. 2001). By this model, shunting a portion of glucose from the circulation into glycogen with subsequent glycogenolysis provides for lactate production by two parallel pathways, traditional glycolysis from glucose and glycolysis from glycogen. Although ATP energy from the glycogen shunt is energetically less efficient than glycolysis from glucose, the shunt allows cell energy storage and glial energy to be provided in milliseconds for rapid neurotransmitter release when needed. Enlarging on their work on cerebral energy needs, Shulman and colleagues expanded the model to include the energetics of working skeletal muscle (Shulman & Rothman, 2001) The hypothesis is a forceful reminder of the immediate role of glycogen over glucose in supplying energy via glycolysis, leading to lactate production in rodents (Brooks & Donovan, 1983; Donovan *et al*. 1983) and humans (Bergman *et al*. 1999 *a*,*b*). The idea of a glycogen shunt is also a reminder that everything, including glycogen, turns over during exercise (Azevedo *et al*. 1998) and that reduced glycogen depletion in exercising muscle is due, in part, to a reduction in turnover.

### Muscle lactate disposal and energy status

Bertocci, Haller and colleagues have used MRS and ^13^C‐lactate tracers to evaluate the role of lactate in muscle energy transduction. By tracking the conversion of ^13^C‐lactate into glutamate in a rat muscle preparation, a TCA cycle intermediate in equilibrium with α‐ketoglutarate, it was confirmed that lactate is readily oxidized by skeletal muscle during rest and contraction, and directly enters the TCA cycle (Bertocci & Lujan, 1999). Also, using ^31^P MRS to study muscle metabolism in patients with McArdle disease, a knock‐out experiment of Nature involving phosphofructokinase (PFK) deficiency, lactate infusion was shown to improve muscle force output and energy status in terms of higher phosphocreatine (PCr) and lower adenosine diphosphate (ADP) and inorganic phosphate (P_i_) levels (Bertocci *et al*. 1993). Thus, by means of exogenous lactate infusion investigators were able to bypass the enzymatic block at PFK. Subsequently, in studies using vascular infusion of [1‐^13^C]lactate and arterial‐venous difference measurements across resting and exercising legs of McArdle disease patients, investigators concluded that ‘lactate formation is mandatory for muscle energy generation during exercise.’ In part those results could have been predicted based on results of studies on electrically stimulated isolated rat muscles in which lactate addition to the incubation bath helped maintain cell membrane chemical‐electrical gradients and ward off fatigue (de Paoli *et al*. 2007).

### Lactate metabolism in injuries and illnesses

This issue has been addressed in recent reviews (Brooks, 2018, 2020 *a*), but there appear to be both positive and negative aspects to lactate metabolism in illnesses and injuries (Table 1).

**Table 1:** **Potential for lactate treatment for illness and injury (from** **Brooks, 2020*a***)

Resuscitation (fluid, electrolytes, energy) (Azevedo *et al*. 2007; Garcia‐Alvarez *et al*. 2014 *a*; Marik & Bellomo, 2016)
Acidosis (exogenous lactate infusion has an alkalotic effect) (Miller *et al*. 2005; Wu *et al*. 2011; Marik & Bellomo, 2016)
Regulation of glycaemia (lactate is the major gluconeogenesis (GNG) precursor) (Meyer *et al*. 1998, 2002; Gerich *et al*. 2001; Marik, 2009)
Traumatic brain injury (lactate is brain fuel and anti‐inflammatory) (Glenn *et al*. 2015 *a*)
Inflammation (via GPR81 binding down stream signalling lactate inhibit the inflammasome) (Hoque *et al*. 2014)
Acute pancreatitis and hepatitis (lactate is an energy substrate, a GNG precursor and anti‐inflammatory agent) (Hoque *et al*. 2014)
Myocardial infarction, cardiac surgery and acute heart failure (lactate is heart fuel) (Shapiro *et al*. 2005; Bergman *et al*. 2009 *b*)
Burns (lactate is an energy substrate, a GNG precursor and anti‐inflammatory agent) (Spitzer, 1979)
Sepsis (lactate incorporation in resuscitation fluids can support maintenance of blood pressure and circulation, and help deliver antibiotics, as well as being an energy substrate, a GNG precursor and have an anti‐inflammatory effect) (Garcia *et al*. 1995; Marik & Bellomo, 2016)
Dengue (lactate is an energy substrate, a GNG precursor and anti‐inflammatory agent) (Wu *et al*. 2011; Somasetia *et al*. 2014)
Cognition (lactate readily crosses the blood‐brain barrier, fuels neurons and stimulates secretion of brain‐derived neurotrophic factor (BDNF), improves executive function and memory) (Rice *et al*. 2002; Holloway *et al*. 2007; Hashimoto *et al*. 2018)
Wound healing (Hunt *et al*. 2007) and muscle regeneration after injury (Tsukamoto *et al*. 2018; Ohno *et al*. 2019).

## Positive effects – lactate or supplemental lactate treatment

Lactate treatment can be involved in resuscitation following environmental stresses (dehydration and acidosis), inflammation (hepatitis and pancreatitis), infectious (Dengue and sepsis) and non‐infectious diseases (hypoglycaemia) and traumatic conditions (brain injury, myocardial infarction, burns and wound healing).

## Negative effects – overabundance of lactate production and accumulation

### Cancer

Seminal studies have shown that lactate production and accumulation occur in cancer cells incubated under fully aerobic condition (Warburg & Minami, 1923, 1927). Subsequently, accelerated glycolysis leading to lactate production in cancer cells has been implicated in many stages in carcinogenesis (San‐Millan & Brooks, 2017). More recently, blocking of MCTs in driver (glycolytic) and oxidative (recipient) tumour cells can minimize lactate exchange, in effect killing both cell types by pickling the drivers and starving the recipients, thus arresting tumour development (Semenza, 2008; Sonveaux *et al*. 2008). To date, however, the use of pharmacological agents to block MCTs in cancer is contraindicated because of the ubiquitous expression of MCTs in most cell types and negative consequences for the host. Additionally, blocking or deleting HCAR‐1 leads to a reduced transcription of cancer‐regulated genes, thus diminishing cancer cell proliferation in animal and cell culture models (Brown *et al*. 2020; Xie *et al*. 2020). At present there are no definitive data indicating that aberrant lactate metabolism causes cancer, but it has been observed that lactate accumulation is associated with upregulation of cancer promoting genes and downregulation of cancer suppressor genes (San‐Millan *et al*. 2020).

Errors in the ability to transport or exclude lactate may have serious consequences. For instance, embryologic deletion of the lactate transporters (monocarboxylate transporters, MCTs) is lethal. For glucoregulation when blood lactate is elevated as in exercise or other conditions, MCTs must be excluded from insertion into pancreatic β‐cell plasma membranes (Pullen *et al*. 2010; Rutter *et al*. 2015). While MCTs are present on plasma and mitochondrial membranes of most cells and tissues, exclusion of MCTs from pancreatic β‐cell plasma membranes keeps extracellular lactate from entering and affecting intracellular redox, thus interfering with glucose sensing and insulin secretion (Bender *et al*. 2006). Noteworthy in this regard is that individuals experiencing failed suppression of pancreatic β‐cell MCT expression become hypoglycaemic during hard exercise leading to lactataemia because the combination of high insulin and increased glucose disposal through metabolism causes profound hypoglycaemia (Otonkoski *et al*. 2007).

## Lactate shuttling into the future: unresolved issues and future directions

The more lactate shuttling is studied, the more is revealed about the expansive nature of lactate metabolism and its central role in physiology. Consider for instance several of the topics discussed above that have been studied separately, but seldom investigated in an integrated fashion. Seemingly, there is need for a more integrative approach to develop models of physiological organization. Seemingly also, we may learn from, or contribute to, research in other fields of science and medicine. Some examples follow.

### Nutrition, digestion, muscle physiology and the microbiome

For instance, consider that the consumption of dietary CHO elicits neuroendocrine signals to splanchnic tissues and brain. In the upper GI tract, entry of glucose for CHO digestion is disposed of via direct and indirect pathways as described in the glucose paradox. Glucose released into the systemic circulation via the hepatic portal vein is taken up by red muscle where glycogen synthesis and glycolysis are stimulated. Subsequently, in the GI tract glucagon‐like peptide‐1 (GLP‐1) and glucose‐dependent insulinotropic polypeptide (GIP) are released from enteroendocrine cells in response to nutrient ingestion and the presence of glucose and lactate. GLP‐1 receptors (GLP‐1Rs) on enteric neurons, including intrinsic afferent neurons, and extrinsic spinal and vagal sensory afferents, have potentially numerous effects including enhancement of insulin secretion and mediation of satiety signalling, via effects on the hypothalamic arcuate and paraventricular nuclei.

It goes without citation that short‐ and long‐term nutrition and blood glucose regulation is heavily tied to hunger and satiety signalling. The biochemistry behind appetite regulation is a complicated and active area of research (Gale *et al*. 2004; Murphy & Bloom, 2006). Suffice it to state that in the arcuate nucleus of the hypothalamus effects of the appetite stimulating (feeding) (neuropeptide y (NPY)) centre are balanced against those of the appetite inhibitory (satiation) [pro‐opio‐melanocortin (POMC)] centre. Hormones that inform the hypothalamic centres that regulate appetite include insulin, ghrelin, leptin, peptide YY (PYY), glucagon like peptide‐1 (GLP‐1) and others (Ghosal *et al*. 2013). Results of classic studies relating physical activity level and diet (Mayer *et al*. 1956), as well as practical experience, indicate that maximal exercise has an appetite inhibiting effect. For instance, few are hungry immediately after maximal exercise to exhaustion. The suppressive effect of lactate on appetite (Schmid *et al*. 2008; Schultes *et al*. 2012; McCarthy *et al*. 2020) is consistent with data showing that lactataemia suppresses ghrelin secretion (Islam *et al*. 2017; Vanderheyden *et al*. 2020). The ghrelin receptor (growth hormone secretagogue receptor (GHSR‐1α)) is a G‐protein coupled receptor expressed throughout both the stomach and GI tract. Recently, it was found that lactate, short chain fatty acids and other bacterial excretions in the GI tract are able to attenuate ghrelin mediat*e*d signalling through the GHSR‐1α (Torres‐Fuentes *et al*. 2019). Hence, in combination with lactate produced by gut microbiota, the high blood lactate of exercise can enter the bowel via sodium‐mediated monocarboxylate transporters (sMCT) and attenuate ghrelin receptor signalling, perhaps revealing how hard exercise attenuates hunger.

Continuing with the course of chyme down the GI tract, microbiota give rise to lactate that is either converted to butyrate, released into the systemic circulation, or excreted (*vide supra*). However, it is clear that more research is needed to better understand how upper and lower bowl processes are regulated and integrated *in vivo*. Consider for instance, the physiology of someone consuming a sports drink during exercise. Notwithstanding the organoleptic effects of cool beverage consumption (Jeukendrup & Chambers, 2010), we would benefit from the development of an overarching model of nutrient consumption and disposal during rest and exercise.

## Metabolic flexibility and lactataemia

Lactataemia limits lipid oxidation by blocking lipolysis and oxidation of FFAs (*vide supra*). From the fields of obesity, diabetes and exercise physiology research come the concepts of metabolic flexibility (Kelley *et al*. 1999) and crossover (Brooks & Mercier, 1994). Metabolic flexibility describes the ability of individuals to switch between key fuel energy substrates in response to changing physiological conditions such as obesity and diabetes. The crossover concept additionally considers the effect of metabolic rate on energy substrate partitioning. With regard to an effect of lactataemia on energy substrate partitioning, Jones and colleagues observed that metabolically inflexible subjects had an increased fasting plasma lactate compared to aged matched controls (Jones *et al*. 2019). Similarly, San‐Millán and Brooks observed that diabetic and obese subjects showed increased circulating lactate and decreased lipid oxidation during rest and exercise compared to age matched lean and highly trained athletes (San‐Millan & Brooks, 2018). Further research is needed to determine the mechanisms by which lactataemia limits metabolic flexibility in sedentary, obese and older individuals. Elaboration of those mechanisms could lead to behavioural and pharmacological treatments for metabolic inflexibility in illnesses and ageing.

### Epigenetic modifications by lactylation of histones and other potential mechanisms

Glis family zinc finger (Glis1) is a transcription factor known to be involved in cell reprogramming, such as transformation of somatic to pluripotent cells. In somatic cells Glis1 binds to glycolysis genes to open them and promote transcription (Li *et al*. 2020). This activates glycolysis, without affecting OXPHOS, thus increasing lactate and acetyl‐CoA levels that result in an increase in histone lactylation and acetylation. Further, histone lactylation in some (Pan Kla and H3K18la), but not all (H3K9me3 and H3K27me3) cultured tumour cells increased substantially after Glis1 overexpression and decreased after Glis1 knockdown. Taken together, the results offer the possibility that Glis1 modulates histone acetylation and lactylation during cell reprogramming. Given these hints in the literature, it may be worthwhile to learn from progress in regenerative medicine and cancer biology to expand our knowledge of the effects of diet and exercise on histone modulation in disease and ageing processes.

### Fertilization *in vivo* and *in vitro*

It is recognized by workers in some fields of science (e.g. sepsis: Marik & Bellomo, 2016) that progress in understanding the biological roles of lactate is attributable to the contributions of exercise physiology summarized above and previously (Brooks, 2018; Ferguson *et al*. 2018). Clearly all can benefit from greater understanding, including those in the fields of *in vitro* fertilization (IVF) in which the classic antagonism between glycolytic and oxidative metabolism is on display. In describing lactate shuttles above, the relationship between lactate secreting Sertoli and sperm cells was described as the ‘primal demonstration’ of cell‐cell lactate shuttling. The ‘good thing, bad thing’ dichotomy of lactate carries over to the field of IVF, which is typically conducted on blastocysts in culture media exposed to air, in which the $P_{O_{2}}$ is 5× greater than *in vivo*. Therefore, the consequences of hyperoxic ROS generation are a major concern because of the potential effects of ROS on mitochondrial morphology and function in the embryos (Belli *et al*. 2020). Similarly, because a disrupted microenvironment can lead to problems in postnatal development (Feuer *et al*. 2017), high lactate and low pH have been matters of concern. Much like the Warburg effect of high rates of aerobic glycolysis in cancer cells and working muscles, mammalian blastocysts exhibit high capacities for aerobic glycolysis and lactate secretion that may create a favourable microenvironment for uterine implantation and invasion. Beyond providing an energy supply, glycolysis in blastocysts creates a microenvironment around the embryo to contribute to the disaggregation of uterine tissues to facilitate trophoblast invasion. Further, as in wound healing, it may be that lactate acts as a signalling molecule to elicit vascular endothelial growth factor (VEGF) recruitment from uterine cells to promote angiogenesis in the uterus. It has also been suggested that the region of high lactate created by the blastocyst modulates the activity of the local immune response, thereby promoting immune tolerance (Gardner, 2015). Moreover, lactate produced during delivery may reduce post‐partum uterine inflammation via the lactate receptor HCAR‐1 (Madaan *et al*. 2017). As discussed above, several sets of independent studies indicate a role of lactate in mitigating inflammation. Because of the role of inflammation in chronic diseases, it seems that focused efforts on the role of lactate in reducing inflammation may be of importance.

## Summary

Time is overdue to turn the page on understanding lactate metabolism and consider lactate shuttling as an important component of intermediary metabolism *in vivo*. Lactate shuttling between producer (driver) and consumer (recipient) cells requires the presence of cell‐cell and intracellular lactate shuttles that fulfil at least three purposes; lactate is: (1) a major energy source, (2) the major gluconeogenic precursor and (3) a signalling molecule. There is little or no evidence for oxygen inadequacy giving rise to lactate production and accumulation in resting or exercising subjects, even in the hypoxia of high altitude (Brooks *et al*. 1991). Rather, there is abundant evidence that lactate production occurs in fully aerobic tissues and organs (Gertz *et al*. 1981; Stanley *et al*. 1985; Richardson *et al*. 1998; Park *et al*. 2015). Recognition for lactate shuttling came first in studies of physical exercise where roles of driver and recipient cells were obvious. However, simultaneously and independently the presence of lactate shuttling as part of postprandial glucose disposal was recognized in studies of lab animals (Foster, 1984) and humans (Woerle *et al*. 2003). Importantly, lactate (not pyruvate) enters the mitochondrial reticulum to support cell energy homeostasis by oxidative phosphorylation of ADP and creatine (Hashimoto *et al*. 2006). Hence, mitochondrial respiration creates the physiological sink for lactate disposal *in vivo*. As research progresses important facets of lactate shuttling are becoming recognized with regard to cell signalling and metabolic regulation (Brooks, 2020*a*). In diverse tissues lactate acts by mass action, cell redox regulation, ROS generation, allosteric binding and lactylation of histones. By inhibiting lipolysis in adipose tissue, via HCAR‐1 binding and CREB activation, and muscle mitochondrial fatty acid uptake, via malonyl‐CoA and CPT1, lactate controls lipid oxidation and overall energy substrate partitioning. Repeated lactate exposure from regular exercise results in adaptive processes such as mitochondrial biogenesis and other healthful circulatory and neurological characteristics such as improved physical work capacity, metabolic flexibility (Brooks, 2018), memory and cognition (Suzuki *et al*. 2011; El Hayek *et al*. 2019). The importance of lactate and lactate shuttling in healthful living is further emphasized when lactate signalling and shuttling are dysregulated as occur in cancer (San‐Millan & Brooks, 2017) and other conditions such as following traumatic brain injury (Glenn *et al*. 2015 *b*), metabolic syndrome (Brooks, 2018; San‐Millan & Brooks, 2018), inappropriate insulin signalling (Rutter *et al*. 2015) and sepsis (Garcia‐Alvarez *et al*. 2014 *a*). While much has been done to determine energetic regulation in steady and transient states of metabolism, the importance of measuring the dynamics of metabolism beyond stagnant use of ‘metabolomics’, by assessing physiological, environmental and age‐related effects on the turnover rates of lactate, glucose, fatty and amino acids as well as structural and metabolic proteins and membranes (i.e. ‘fluxomics’), are research challenges for the new millennium.

Like a phoenix, lactate has again risen to major importance in 21st century biology.

## Additional information

### Competing interests

The authors have no competing interests.

### Author contributions

All authors contributed to writing of the paper and approve of the final version. All persons designated as authors qualify for authorship, and all those who qualify for authorship are listed.

### Funding

Supported by NIH 1 R01 AG059715‐01, Pac‐12 Conference Grant No. 3‐02‐Brooks‐17 and the UCB Centre for Research and Education on Aging (CREA) to G.A.B. There are no other funding sources.
